# Key patient demographics shape innate immune topography in noncritical hypoxic COVID-19 pneumonia

**DOI:** 10.1172/jci.insight.166110

**Published:** 2023-08-22

**Authors:** Allison C. Billi, Rachael Wasikowski, Feiyang Ma, Srilakshmi Yalavarthi, Claire K. Hoy, Yu Zuo, Matthew T. Patrick, Neha Shah, Christine Parker, Chad Aaronson, Alyssa Harbaugh, Matthew F. Lucido, Kerby Shedden, Krishna Rao, Heidi B. IglayReger, Charles F. Burant, J. Michelle Kahlenberg, Lam C. Tsoi, Johann E. Gudjonsson, Jason S. Knight, Yogendra Kanthi

**Affiliations:** 1 Department of Dermatology,; 2 Division of Rheumatology, Department of Internal Medicine,; 3 Division of Cardiovascular Medicine, Department of Internal Medicine,; 4 A. Alfred Taubman Medical Research Institute,; 5 Division of Infectious Disease, Department of Internal Medicine,; 6 Department of Internal Medicine,; 7 Department of Nutritional Sciences,; 8 Department of Computational Medicine and Bioinformatics, and; 9 Department of Biostatistics, University of Michigan, Ann Arbor, Michigan, USA.; 10 Laboratory of Vascular Thrombosis and Inflammation, National Heart, Lung, and Blood Institute, NIH, Bethesda, Maryland, USA.

**Keywords:** COVID-19, Immunology, Innate immunity

## Abstract

Risk of severe disease and death due to COVID-19 is increased in certain patient demographic groups, including those of advanced age, male sex, and obese body mass index. Investigations of the biological variations that contribute to this risk have been hampered by heterogeneous severity, with immunologic features of critical disease potentially obscuring differences between risk groups. To examine immune heterogeneity related to demographic risk factors, we enrolled 38 patients hospitalized with clinically homogeneous COVID-19 pneumonia — defined as oxygen saturation less than 94% on room air without respiratory failure, septic shock, or multiple organ dysfunction — and performed single-cell RNA-Seq of leukocytes collected at admission. Examination of individual risk factors identified strong shifts within neutrophil and monocyte/dendritic cell (Mo/DC) compartments, revealing altered immune cell type–specific responses in higher risk COVID-19 patient subgroups. Specifically, we found transcriptional evidence of altered neutrophil maturation in aged versus young patients and enhanced cytokine responses in Mo/DCs of male versus female patients. Such innate immune cell alterations may contribute to outcome differences linked to these risk factors. They also highlight the importance of diverse patient cohorts in studies of therapies targeting the immune response in COVID-19.

## Graphical abstract

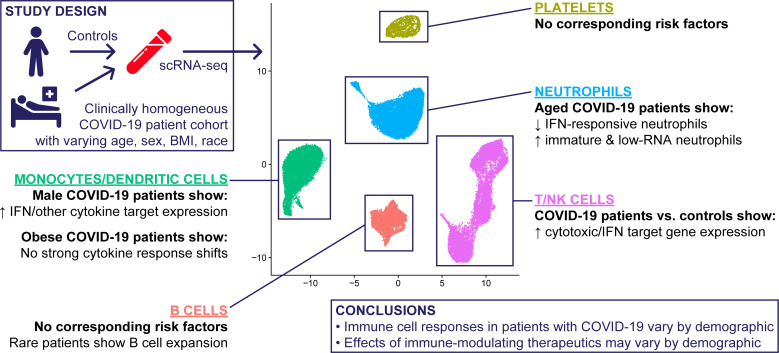

## Introduction

COVID-19, the disease caused by SARS-CoV-2, continues to impose a terrible burden on populations and health systems worldwide. In the early phase of the pandemic, several demographic risk factors emerged as associated with severe disease outcomes, including increasing age (1–5), male biological sex (1, 3, 6), and obesity (7–9). The biological basis of increased risk related to these key patient covariates remains incompletely understood. There is rising concern about inadequate consideration of these patient covariates in COVID-19 study design and outcome reporting (10–13). Additionally, despite targeting a broad spectrum of host defense processes, many COVID-19 therapeutic trials have yielded disappointing results (14, 15). Identification of relevant immunological differences among these demographic groups of patients with COVID-19 may meaningfully inform clinical trial design.

Previous studies (16–20) have revealed shifts in immune cell populations and gene expression signatures that are particularly pronounced in critical disease and might thus obscure variations attributable to other factors. Therefore, to identify risk factor–related variation in immune responses, we enrolled a cohort of 38 patients hospitalized with hypoxic COVID-19 pneumonia of relatively uniform clinical severity and performed single-cell RNA-Seq (scRNA-Seq) on leukocytes isolated from whole blood collected within a day of admission.

Comparison of patient subgroups in the context of key demographic risk factors for severe COVID-19 infection — advanced age (65 years or older), male sex, and obesity (BMI 30.0 or higher) — revealed intriguing cell lineage–specific effects among the major leukocyte types. Whereas transcriptomic differences between patients with COVID-19 and controls were strongest in T/NK cells, most COVID-19 risk factor subgroups showed higher divergence in other leukocyte cell types, varying across the comparisons. We observed prominent transcriptomic shifts in the innate arm of the immune system, with strong neutrophil differences in the age comparison and monocyte/dendritic cell (Mo/DC) differences in the sex and BMI comparisons. This analysis reveals that particular risk factors for severe COVID-19 infection are associated with divergent leukocyte gene expression patterns that suggest heterogeneous immune responses. These findings underscore the importance of representing diverse demographic groups in COVID-19 therapeutic trials, particularly those that target the actions of distinct leukocyte subsets.

## Results

### A cohort of patients hospitalized with clinically homogeneous hypoxic COVID-19 pneumonia.

**Figure 1:**
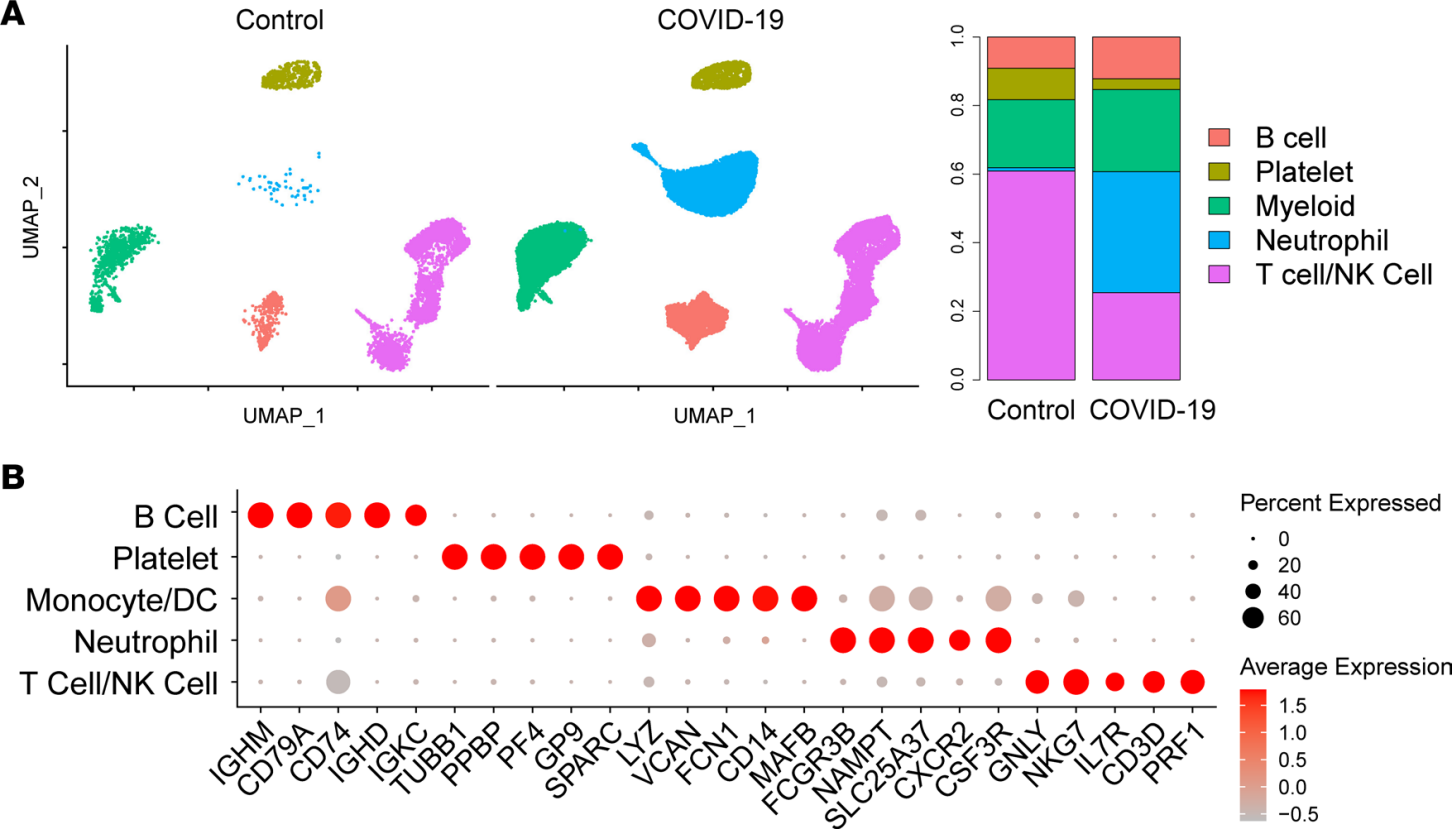
A leukocyte single-cell atlas of a clinically uniform cohort of patients with COVID-19. (**A**) Uniform manifold approximation and projection (UMAP) plot of 35,932 cells colored by cell type and split by disease state. Bar plot, cell type proportions split by disease state. (**B**) Dot plot of representative marker genes for each cell type. Color scale, average marker gene expression. Dot size, percentage of cells expressing marker gene.

Thirty-eight patients presenting to the University of Michigan hospital and 3 healthy controls were enrolled in the study (Table 1 and Supplemental Table 1; supplemental material available online with this article; https://doi.org/10.1172/jci.insight.166110DS1). Patients aged 18 and older were eligible to participate if they were hospitalized with confirmed SARS-CoV-2 infection by a PCR-based assay and required supplemental oxygen at the time of enrollment based on oxygen saturation less than 94% on room air. Patients were not eligible if they exhibited respiratory failure requiring invasive mechanical ventilation, septic shock, or multiple organ dysfunction at enrollment or received cytokine inhibitory therapy (targeting IL-6, IL-6R, IL-1, or Janus kinase). To capture both PBMCs and neutrophils, buffy coats were isolated from whole blood and subjected to scRNA-Seq on the Chromium (10x Genomics) platform. The resulting quality-controlled leukocyte atlas contained 35,932 cells. After batch correction (21), cells were clustered using the R package Seurat and annotated based on canonical cell type marker genes. We identified 5 broad cell types: neutrophils, Mo/DCs, T/NK cells, B cells, and platelets (Figure 1, A and B). All cell types included cells from both patients with COVID-19 and healthy control samples, although proportions of neutrophils were expanded and T cells contracted in COVID-19 samples (Figure 1A and Supplemental Table 2).

We performed subclustering of the 5 major immune cell types detected in our analysis. For each cell type, we compared the transcriptomes of cells from patients with COVID-19 and healthy controls to identify differentially expressed genes (DEGs) and used the count of DEGs exhibiting adjusted *P* values less than or equal to 0.05 and greater than or equal to a 1.2-fold change as a proxy for transcriptomic variation. We adjusted for 4 major covariates that have been shown to affect COVID-19 outcomes: age, sex, BMI, and self-identified race (1–9, 22, 23). This analysis revealed leukocyte transcriptomic differences between patients with COVID-19 and healthy controls most pronounced in T cells and Mo/DCs (Table 2 and Supplemental Table 3). Low neutrophil recovery from our 3 control patients (*N* = 45) (Supplemental Table 2) precluded meaningful calculation of DEGs in comparison with patients with COVID-19.

To explore how patient characteristics contribute to variation in immune responses to COVID-19, we divided the samples of patients with COVID-19 into risk factor subgroups for age, sex, and obesity. Due to the insufficient representation of non-White race patients and the absence of Hispanic patients in our cohort, dedicated analyses of the covariates of race and ethnicity were not possible; however, given the inclusion of Black patients in our cohort and the significance of this risk factor, adjustment for race was still performed. We then tabulated DEGs for each cell type in each risk factor subgroup comparison while adjusting for the 3 other major covariates. The most divergent cell types varied across these analyses (Table 2). To further understand these subgroup-related differences, we proceeded with dedicated analyses of highly divergent cell types in each of these comparisons.

### Neutrophils from aged patients with COVID-19 are distinguished by an attenuated IFN response signature.

When comparing patients with COVID-19 65 years of age and older (*n* = 17) to patients younger than 65 years of age (*n* = 21), neutrophils showed the highest number of DEGs (Table 2). Inspection of the 15 most downregulated DEGs (Supplemental Figure 1A) revealed reduced expression of multiple IFN-stimulated genes (ISGs) (*IFITM3*, *IFIT1*, *TNFSF10*, *IFIH1*) in neutrophils of aged patients. The top downregulated DEG was *CLEC12A*, an inhibitory receptor that downmodulates neutrophil activation (24, 25) and amplifies type I IFN responses in BM-derived DCs (26). The 15 top upregulated DEGs included multiple mitochondrial transcripts (*MT-CYB*, *-CO1*, *-CO2*, *-CO3*, and *-ND3*) (Supplemental Figure 1A), suggestive of increased stress (27) or neutrophil immaturity (17).

For a more holistic understanding of transcriptional differences between neutrophils from aged and young patients with COVID-19, we performed upstream regulator analysis using Ingenuity Pathway Analysis (IPA, QIAGEN). This analysis seeks to identify upstream transcriptional regulators that can explain observed gene expression changes based on a database of expected effects between transcriptional regulators and their target genes gleaned from the literature. Upstream regulator analysis can predict which transcriptional regulators might be involved in gene expression changes and whether these regulators are likely activated or inhibited. Ribosomal transcripts were filtered out of the DEG lists before analysis, as these tended to vary en bloc; the same was done for mitochondrial transcripts. Significant results (*P* < 0.05) were filtered for cytokines with scores suggesting either significant activation (*z* score ≥ 2) or significant inhibition (*z* score ≤ –2) in aged neutrophils (Supplemental Figure 1B). The cytokine IL-4 showed the highest activation score in the neutrophils of aged compared with young patients with COVID-19. IL-4 inhibits neutrophil effector functions by restricting expansion and attenuating migration into tissues via downregulation of *CXCR2* (28), which was significantly downregulated (*P* = 5.00 × 10^–25^) in the neutrophils of aged patients with COVID-19 in our data set. Only type I IFNs (IFN-α2, IFN-β1, and IFN-ε) showed significant inhibition scores, consistent with ISG depletion in neutrophils from aged patients with COVID-19 among the DEGs (Supplemental Figure 1A). Together, analyses of age-biased DEGs in patients with COVID-19 suggested neutrophils of aged patients could have altered functional capacity, with attenuation of the transcriptional IFN responses that are activated in normal antiviral modules.

### Aged patients show expansion of immature neutrophils and failure to expand ISG-high neutrophils during COVID-19 infection.

**Figure 2:**
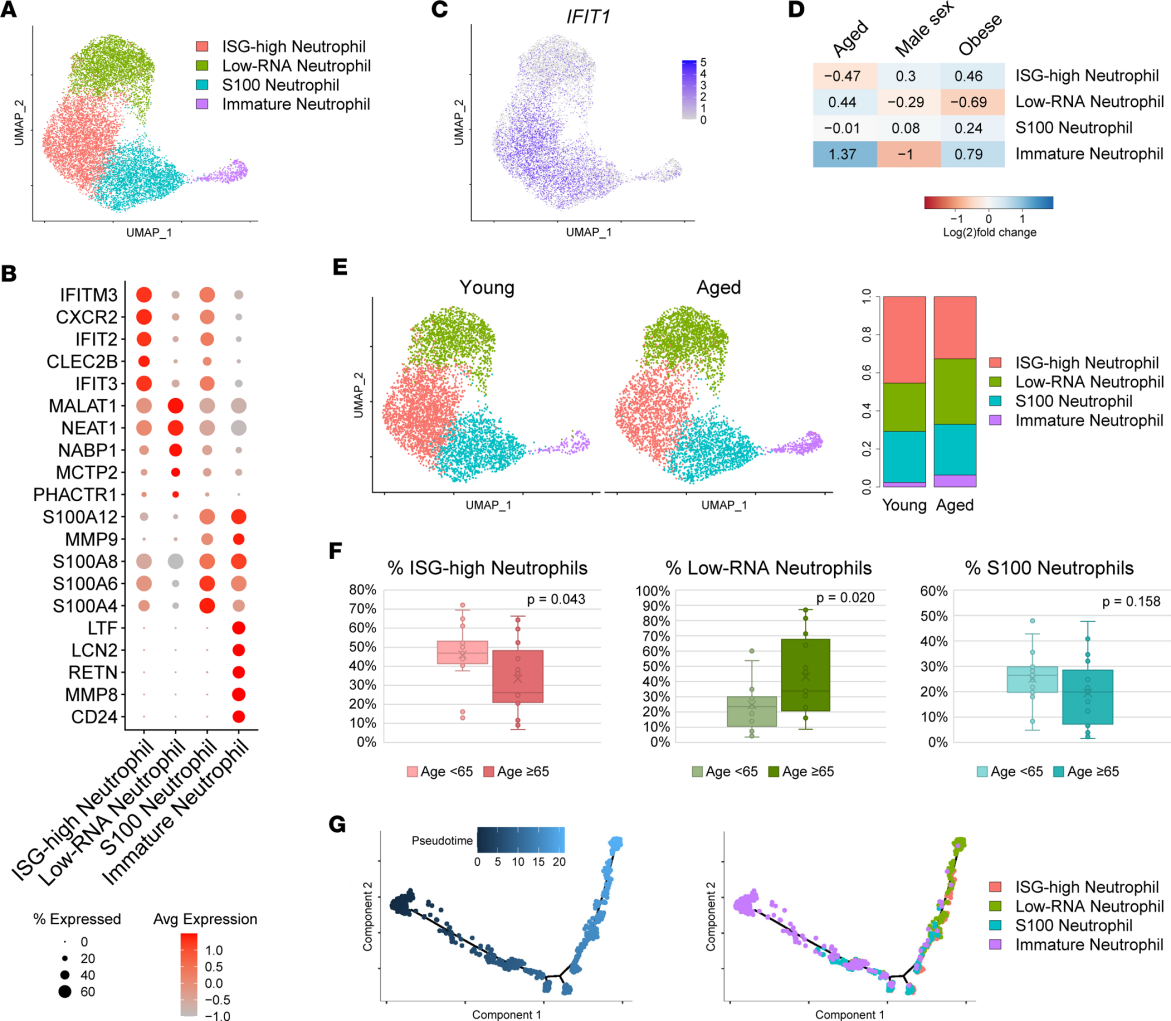
Effects of risk factors on neutrophil shifts in COVID-19. (**A**) UMAP plot of 11,023 neutrophils colored by subcluster. (**B**) Dot plot of representative marker genes for each neutrophil subcluster. Color scale, average marker gene expression. Dot size, percentage of cells expressing marker gene. (**C**) Feature plot of representative IFN-stimulated gene *IFIT1* showing normalized expression values. (**D**) Heatmap of relative expansion or contraction of each neutrophil subcluster in each risk factor subgroup comparison (excluding control patients). Blue, increased in the higher risk subgroup (aged, male sex, obese). Red, increased in the lower risk subgroup (young, female sex, normal BMI). Value shows log_2_ ([number of cells in higher risk subgroup]/[total number of cells in both subgroups]). (**E**) UMAP plot of neutrophils of patients with COVID-19 split by age group. Bar plot, proportion of neutrophils in each subcluster split by age group. (**F**) Box-and-whisker plots of percentages of each of the 3 mature neutrophil subclusters in patients with COVID-19. Box plots show the interquartile range (box), median (line), and minimum and maximum (whiskers). *P* values are shown for 2-tailed heteroscedastic Student’s *t* test. (**G**) Pseudotime plots of neutrophils from all patients with COVID-19 and controls colored by pseudotime (left) and subcluster (right).

To further investigate the source of the divergent ISG signal and other DEGs emerging from the age comparison, we examined the neutrophil subclustering analysis in detail. Four subclusters were identified (Figure 2, A and B). We annotated the smallest subcluster as immature neutrophils based on the expression of markers such as *LTF*, *LCN2*, and *CEACAM8* and the remainder as mature neutrophils. The largest mature neutrophil subcluster was distinguished by the expression of numerous ISGs, including IFITM3 and IFIT1/2/3 (Figure 2, B and C). This subcluster was therefore annotated as ISG-high neutrophils, consistent with a population previously identified by Combes et al. to distinguish mild/moderate from severe COVID-19 (20). The next subcluster comprised neutrophils with lower RNA content and was marked by high expression of long noncoding RNAs (lncRNAs) *MALAT1* and *NEAT1*. The final subcluster showed increased expression of *S100* genes. Insufficient recovery of neutrophils from healthy control samples precluded meaningful comparison with the neutrophils from patients with COVID-19 (Supplemental Table 2).

We next divided patients with COVID-19 into risk factor subgroups and calculated the proportions of the 4 neutrophil subclusters across these comparisons, uncovering distinct neutrophil subcluster profiles across the higher risk groups (Figure 2D). Compared to young patients, aged patients showed expansion of immature neutrophils (Figure 2, D and E). Aged patients further showed a shift in the mature neutrophil population away from ISG-high neutrophils and toward low-RNA neutrophils (Figure 2, D–F), with percentages of these 2 mature neutrophil subsets differing significantly by age (*P* = 0.043, 0.020, and 0.158 for ISG-high, low-RNA, and S100 neutrophils, respectively). To investigate the developmental relationships of the 3 mature neutrophil subsets, we performed pseudotime characterization of all neutrophils in our analysis (Figure 2G). This revealed a single primary pseudotime trajectory spanning from immature to mature neutrophils. S100-high neutrophils appeared more immature, whereas ISG-high and low-RNA neutrophils were located later in pseudotime, corroborating a similar analysis by Combes et al. (20). The extensive pseudotime overlap between the terminal ISG-high and low-RNA neutrophil subclusters suggests that in COVID-19 infection, neutrophils mature into either an ISG-high or an ISG-low, transcriptionally quiescent terminal state. The latter state is overrepresented among neutrophils of aged patients with COVID-19.

### ISG-high neutrophils are intact in male and obese subgroups of patients with COVID-19.

We then assessed whether the neutrophil shifts observed in aged patients with COVID-19 were specific to this risk subgroup or a hallmark of all higher risk subgroups in this analysis. Interestingly, male patients showed an opposite pattern, with expansion of ISG-high neutrophils and contraction of low-RNA and immature neutrophils relative to female patients (Figure 2D). Obese patients also showed expansion of ISG-high neutrophils and contraction of low-RNA neutrophils relative to normal BMI patients (Figure 2D); however, unlike for age, differences in proportions of these neutrophil subsets were not significant for sex or BMI (data not shown). Older age was the only risk factor analyzed that was associated with contraction of ISG-high neutrophils, reflecting a shift of mature neutrophils toward an ISG-low terminal differentiation state. The absence of a universal neutrophil signature associated with higher risk subgroups indicates that whether and how neutrophil transcriptional shifts might predict or influence COVID-19 outcomes could vary by risk factor. It is noteworthy that neutrophils emerged as the most transcriptionally divergent subset only in the age comparison (Table 2). While this data set cannot be used to link shifts in neutrophil phenotype to outcomes, it is possible that these neutrophil shifts may be pathogenic and contribute to age-associated outcome differences in COVID-19.

### Male patient Mo/DC transcriptomes demonstrate enhanced activation scores for IFN and other cytokines.

We next considered the effect of sex as a covariate. Comparison of leukocytes from male (*n* = 26) versus female (*n* = 12) patients with COVID-19 revealed the highest transcriptional divergence in Mo/DCs (Table 2). Examination of the top DEGs enriched and depleted in male versus female COVID-19 Mo/DCs (Supplemental Figure 2A) revealed appropriate recovery of Y-linked genes (*DDX3Y*, *EIF1AY*, and *UTY*) and *XIST*, which enables X chromosome inactivation and, thus, is increased in female cells. Genes enriched in male Mo/DCs relative to female Mo/DCs included multiple ISGs (*IFI27*, *IFI6*, and *ISG15*), prompting us to perform an upstream regulator analysis to identify cytokines predicted to promote sex-biased Mo/DC gene expression patterns in patients with COVID-19. Numerous cytokines showed significant activation scores in male Mo/DCs relative to female Mo/DCs, including IFNs, TNF, IL-6, and IL-1β, whereas only the IL-1 receptor antagonist (IL1RN) showed a significant inhibition score relative to female Mo/DCs (Supplemental Figure 2B).

**Figure 3:**
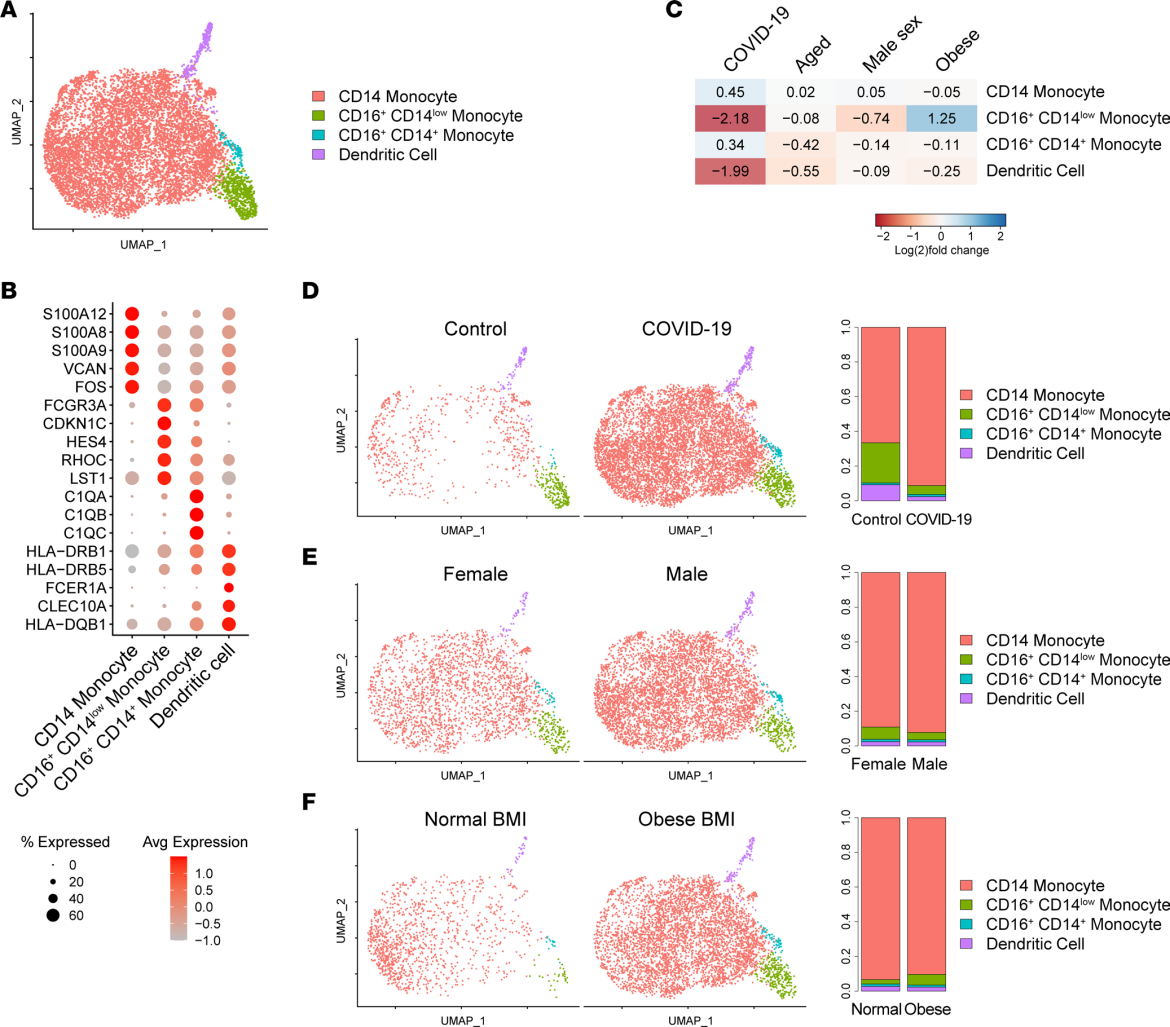
Effects of risk factors on Mo/DC shifts in COVID-19. (**A**) UMAP plot of 8,420 Mo/DCs colored by cell type. (**B**) Dot plot of representative marker genes for each Mo/DC type. Color scale, average marker gene expression. Dot size, percentage of cells expressing marker gene. (**C**) Heatmap of relative expansion or contraction of each Mo/DC type in comparison of patients with COVID-19 versus controls (disease column) and in each risk factor subgroup comparison (excluding control patients). Blue, increased in the higher risk subgroup (aged, male sex, obese). Red, increased in the lower risk subgroup (young, female sex, normal BMI). Value shows log_2_ ([number of cells in higher risk subgroup]/[total number of cells in both subgroups]). (**D**) UMAP plot of Mo/DCs split by disease state. Bar plot, proportion of Mo/DCs in each subcluster split by disease state. (**E**) UMAP plot of Mo/DCs of patients with COVID-19 split by sex. Bar plot, proportion of Mo/DCs in each subcluster split by sex. (**F**) UMAP plot of Mo/DCs of patients with COVID-19 split by BMI group. Bar plot, proportion of Mo/DCs in each subcluster split by BMI group.

We then performed Mo/DC subclustering. Subclusters were identified and annotated as CD14^+^ Mos (corresponding to classical Mos), CD16^+^CD14^lo^ (nonclassical) Mos, CD14^+^CD16^+^ (intermediate) Mos, and DCs based on their expression of *CD14*, *FCGR3A* (encoding CD16), and MHC class II transcripts (Figure 3, A and B). We first compared COVID-19 versus control patient cells, validating the anticipated contraction of CD16^+^CD14^lo^ Mos and DCs (16) (Figure 3, C and D). Comparison of male versus female patients with COVID-19 revealed contraction of CD16^+^CD14^lo^ Mos in men, contrary to a prior report (29), with proportions of other Mo/DC subtypes otherwise similar (Figure 3, C and E). Unlike for neutrophils, subclustering did not identify discrete populations of ISG-high cells; rather, top ISGs upregulated in COVID-19 versus control Mo/DCs mapped diffusely across the population (data not shown). Deeper analysis of sex differences within CD14^+^ Mos, which constituted the vast majority of Mo/DCs, revealed that CD14^+^ monocyte sex DEGs (Supplemental Figure 2C) overlapped highly with the sex DEGs identified in the full Mo/DC analysis above (Supplemental Figure 2A), including demonstration of ISG upregulation in male patients relative to female patients.

Together, our analysis of biological sex in patients with COVID-19 revealed the most pronounced effects in the Mo/DC compartment, where men exhibited significantly higher activation scores for many cytokines, including TNF and IL-1β, and the most powerful discriminating transcriptomic signal was profound enhancement of IFN response scores in Mo/DCs of men relative to women.

### BMI comparison reveals only modest evidence of enhanced cytokine responses in Mo/DCs of obese patients with COVID-19.

We next considered the covariate of BMI. Comparison of obese (*N* = 23) versus normal BMI (*N* = 6) patients also highlighted Mo/DCs as the most transcriptionally divergent cell type (Table 2). Examination of the top DEGs enriched and depleted in Mo/DCs of patients with COVID-19 with obese versus normal BMIs (Supplemental Figure 2D) revealed an interesting contrast to the sex analysis (Supplemental Figure 2A). While both higher risk groups (obese and male) showed upregulation of *CXCL8*, top DEGs otherwise showed a largely reciprocal enrichment pattern, with markers of the higher risk group in 1 comparison resembling those of the lower risk group in the other (i.e., obese resembled female and normal BMI resembled male). This included several ISGs that were upregulated in both male and normal BMI patients. For further context, we performed upstream regulator analysis of Mo/DC BMI DEGs via IPA (Supplemental Figure 2E). Unlike in the sex comparison, very few cytokines showed significant activation scores in Mo/DCs from obese patients with COVID-19 relative to those with normal BMI. IL-1β was again activated, as was IFN-γ, albeit with a much lower activation score than in the sex comparison due to the presence of many ISGs among both increased and decreased genes.

The Mo/DC subclustering analysis revealed further dissimilarity between sex and BMI comparisons, with obese patients showing expansion of CD16^+^CD14^lo^ Mos (Figure 3, C and F). Thus, while both obesity and male biological sex predispose patients to more severe COVID-19, obese patients do not show the same strong activation scores for IFN and other cytokines in Mo/DCs or Mo subset shifts as male patients in comparison with their respective counterparts.

### T/NK cell profiles during COVID-19 infection show subtler variation across risk factor subgroups.

DEG counts suggested that T/NK cells were not the most transcriptionally divergent immune cell type in age, sex, or BMI comparisons; however, T/NK cells did show the highest DEG count in a comparison of patients with COVID-19 and controls (Table 2). Further analysis of T/NK cells in patients with COVID-19 versus controls broadly corroborated prior findings. T/NK cells were overall contracted in patients with COVID-19 versus controls (Figure 1A). Top DEGs enriched in the T/NK cells of patients with COVID-19 included ISGs and genes encoding cytotoxic granular proteins and S100s (Supplemental Figure 3A). Upstream regulator analysis showed strong activation scores for many cytokines, including IFNs, TNF, and IL-1β, in the T/NK cells of patients with COVID-19 (Supplemental Figure 3B).

**Figure 4:**
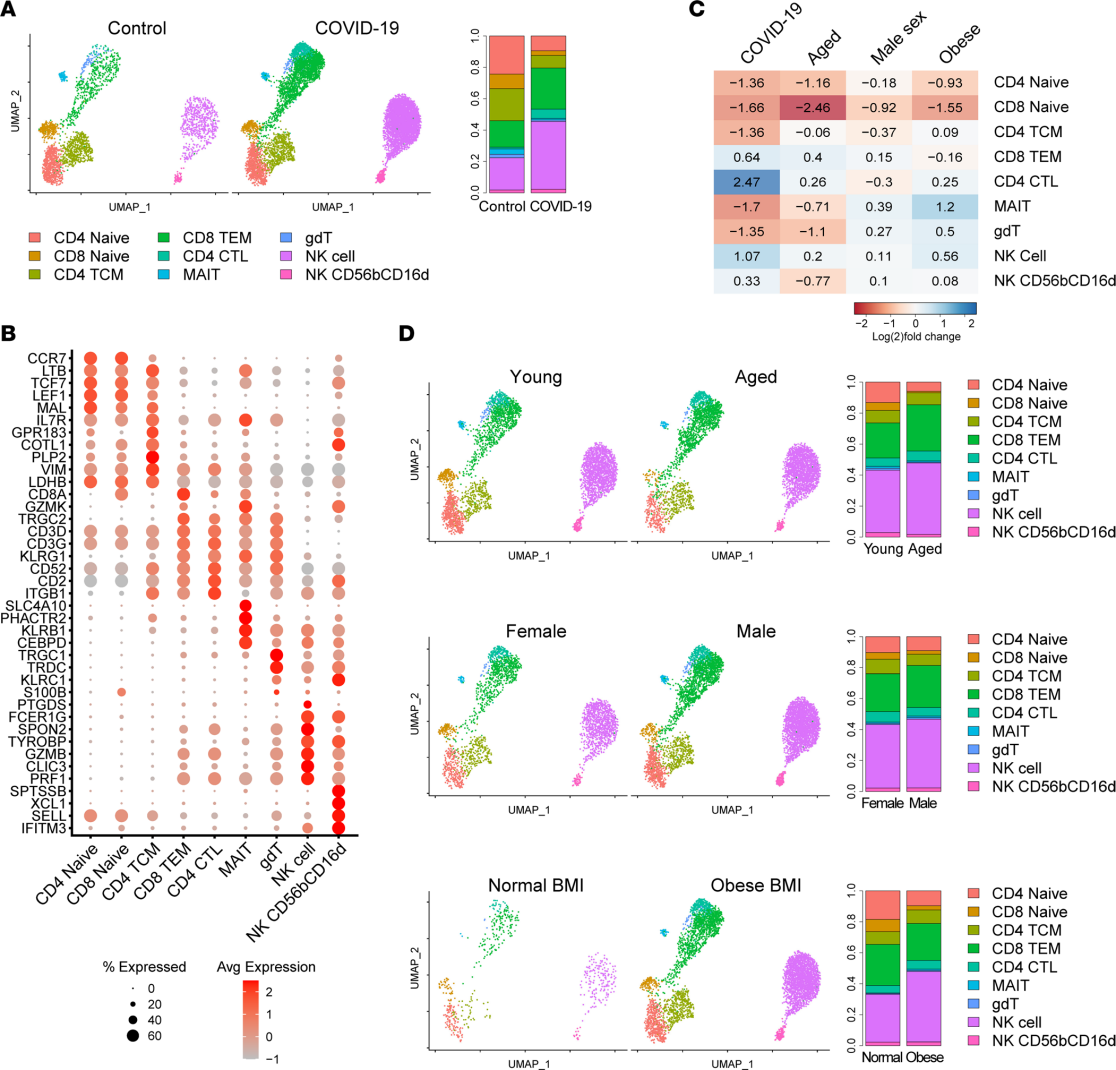
Effects of risk factors on T/NK cell shifts in COVID-19. (**A**) UMAP plot of 10,839 T/NK cells colored by cell type and split by disease state. Bar plot, proportion of T/NK cells in each subcluster split by disease state. (**B**) Dot plot of representative marker genes for each T/NK cell subset. Color scale, average marker gene expression. Dot size, percentage of cells expressing marker gene. (**C**) Heatmap of relative expansion or contraction of each T/NK cell type in comparison of patients with COVID-19 versus controls (disease column) and in each risk factor subgroup comparison (excluding control patients). Blue, increased in the higher risk subgroup (aged, male sex, obese). Red, increased in the lower risk subgroup (young, female sex, normal BMI). Value shows log_2_ ([number of cells in higher risk subgroup]/[total number of cells in both subgroups]). (**D**) UMAP plots and bar plots of T/NK cells split by risk factor subgroup. Only cells of patients with COVID-19 from the indicated risk factor subgroups are presented on each plot. MAIT, mucosal-associated invariant T.

T/NK cell subclustering and annotation demonstrated the presence of all T cell subsets in both controls and patients with COVID-19 (Figure 4, A and B). Comparison of the T/NK cell subsets of patients with COVID-19 and controls revealed relative expansion of CD4^+^ T cells with cytotoxic activity (CD4^+^ CTLs), CD8^+^ effector memory T cells (TEM), NK cells, and CD56^bright^CD16^dim^ NK cells in patients with COVID-19 (Figure 4C). This increased representation of cytotoxic T/NK cell types likely contributed to the emergence of genes encoding cytotoxicity factors among the top T/NK cell COVID-19–enriched DEGs (Supplemental Figure 3A). However, prior work suggests the actual cytotoxic function of these cells in severe COVID-19 is likely impaired (30). In the risk factor subgroup comparisons, T/NK cell shifts in age and sex comparisons overall showed many similarities with the comparison of patients with COVID-19 and controls (Figure 4, C and D). The BMI comparison included very few cells in the normal BMI subgroup, limiting interpretability. Given that the risk factor subgroups show less transcriptomic divergence within T/NK cells than other major immune cell types, shifts within T/NK cell subsets may be less likely to underlie the risk factor outcome differences.

### B cell expansion in COVID-19 is driven by outliers and not predicted by the examined risk factors.

During preliminary subclustering for quality control prior to DEG calculation (Supplemental Figure 4, A and B), B cells were the only major cell type to show numerous subclusters composed almost exclusively of cells from 3 patients with COVID-19 (Supplemental Figure 4C). Several of these clusters showed high expression of plasmablast markers such as *CD27* and genes encoding the constant regions of IgA and IgG (Supplemental Figure 4, A and B), suggesting a function in secretion of antigen-specific Abs. In particular, subcluster 5 expressed *XBP1*, *POU2AF1*, and *IRF4*, consistent with the *XBP1^+^* plasma cells found to be significantly expanded in some patients with COVID-19 (31, 32). Only 2 B cell subclusters showed typical donor diversity (Supplemental Figure 4C), and no B cells were identified for a substantial portion of the patients (15 of 38, 39%) (Supplemental Table 5). Subcluster 1 was identified as naive B cells based on the expression of markers such as *TCL1A* and *IGHD*, while subcluster 4 was identified as memory B cells based on expression of *MS4A1* (Supplemental Figure 4B). In preliminary subclustering of neutrophils, Mo/DCs, or T/NK cells for quality control, no similar patient outliers were identified.

Due to their highly skewed B cell profiles, B cells deriving from the 3 outlier donors were excluded from DEG calculations. DEG counts did not identify B cells as the most transcriptionally divergent leukocyte type for any subgroup comparison (Table 2). Of note, none of the major demographic risk factors examined in this study was shared by all 3 outliers (Supplemental Table 1 and Supplemental Figure 4D). This suggests that other factors may influence B cell shifts more strongly than the covariates of age, sex, BMI, or race.

### Platelet transcriptomes vary minimally among the examined risk factor subgroups of patients with COVID-19.

Multiple reports have implicated platelets in COVID-19 disease severity by examining patient platelet transcriptomes using bulk RNA-Seq (33–35). We therefore also investigated platelets as potential contributors to variation among the risk factor subgroups of patients with COVID-19. While comparison of healthy controls and COVID-19 patient platelet transcriptomes revealed 126 DEGs, platelet DEG numbers for risk factor subgroup comparisons were far fewer than for any other cell type examined (Table 2), suggesting transcriptomic homogeneity among subgroups.

## Discussion

Here, we present the analysis of scRNA-Seq data from 38 patients hospitalized for hypoxic COVID-19 pneumonia with clinically homogeneous disease at presentation. We exploited the clinical consistency of our cohort to examine the leukocyte transcriptome shifts associated with demographic risk factors for severe COVID-19. Covariate associations were most pronounced among innate immune cells. Age showed the strongest effects in neutrophils, whereas sex and BMI showed the strongest effects in Mo/DCs (Table 2). Surprisingly, immune cell profiles were not consistent across higher risk subgroups; for example, aged patients with COVID-19 showed loss of ISG-high neutrophils, whereas male and obese patients showed expansion of ISG-high neutrophils (Figure 2D). While our deliberate selection of a cohort with clinically homogeneous disease limits our ability to associate these immune cell profiles with clinical outcomes, our results reveal the critical importance of diverse patient inclusion in COVID-19 studies. This is particularly essential considering that many of the drugs investigated for treatment of COVID-19 have targeted the immune response rather than the virus itself (36). Our findings further suggest that extrapolation of clinical trial results to demographic groups not represented in such trials should be done with caution and could result in negative patient outcomes.

In our study, aged patients with COVID-19 showed expansion of circulating immature neutrophils and contraction of ISG-high neutrophils (Figure 2D). Abnormal neutrophil populations in COVID-19 have been documented to exhibit immature characteristics, consistent with emergency granulopoiesis (16, 18, 19). This shift toward immaturity is associated with a negative prognosis (16, 18, 19) and may even contribute to the suppression of T cell proliferation in patients with severe COVID-19 (37). It is unclear how contraction of ISG-high neutrophils among aged patients with COVID-19 might influence disease outcomes. ISG-high neutrophils have been reported in multiple COVID-19 studies (18, 20, 37) and were identified by Combes et al. as the only neutrophil subset distinguishing mild/moderate from severe disease (20), suggesting a protective effect. Indeed, multiple studies have found defective IFN responses in patients with severe COVID-19 (20, 38–43), although there are also a few reports of excessive IFN responses being associated with worse patient outcomes (44, 45). ISG-high neutrophils were also marked by prominent expression of *CXCR2* (*P* < 2.23 × 10^–308^) (Figure 2A) and *CXCR1* (9.27 × 10^–69^) (data not shown), which encode receptors for IL-8/CXCL8 and related chemokines that enable trafficking to inflamed tissue. Whether this enhances capacity for disease defense or pathologic tissue damage remains to be determined.

In aged patients, reduced numbers of ISG-high neutrophils were balanced by expansion of the low-RNA neutrophils that demonstrated high expression of lncRNAs *NEAT1* and *MALAT1*. Several populations of mature neutrophils marked by *NEAT1* were also identified by Combes et al. that did not differ significantly in proportion between mild/moderate and severe disease; however, in that same study, neutrophil *NEAT1* and *MALAT1* were among the most significantly increased genes tracking in patients with severe versus mild/moderate COVID-19 (20). A prior study utilizing scRNA-Seq to investigate neutrophils demonstrated that the gene number and total unique molecular identifiers (UMIs) both increase in neutrophils during bacterial infection, consistent with increased transcriptional activity (17); thus, low-RNA neutrophils may indeed represent a less active mature neutrophil subset. Further, weak expression of *CXCR2* compared with ISG-high neutrophils suggests reduced capacity of low-RNA neutrophils for migration into inflamed tissues. Of note, S100-high neutrophils, reported to be anticorrelated with ISG-high neutrophils and correspond to a severe fate (20), were equally represented in aged and young patients with COVID-19 (Figure 2, D–F). Together, our age analysis suggests that during COVID-19 infection, neutrophils of aged patients are less likely to mature into ISG-high neutrophils, instead entering an alternative terminal differentiation state characterized by low-RNA expression and lacking putative antiviral properties conferred by ISG upregulation.

In our analysis of sex, the Mo/DCs of male patients with COVID-19 showed enhanced cytokine response scores, including IFN-induced gene expression changes. This was interesting given that robust ISG expression in the mononuclear phagocyte pool has been reported to be associated with mild COVID-19 (20). Specific DEGs emerging from this analysis were suggestive of the male sex conferring increased risk. Male Mo/DCs showed upregulation of *SELL*, encoding CD62L/L-selectin, and *PLAC8*, a prematuration marker that distinguishes a sepsis-associated immature monocyte state (46); both were previously identified as markers of a Mo population detected almost exclusively in patients with severe COVID-19 versus those with mild disease or healthy controls (18). Furthermore, baricitinib, a JAK1/2 inhibitor with IFN-blocking activity, has shown benefits in several therapeutic trials (47–49); however, in a study demonstrating its effectiveness in combination with remdesivir in reducing time to recovery from COVID-19, the effect was significant among men but not women (48). In addition to highlighting the importance of stratified analyses in COVID-19 therapeutic trials, this sex difference in the therapeutic benefit of JAK inhibition could represent a clinical corollary for the enhanced IFN activation signature we observed in Mo/DCs of male patients with COVID-19.

While various cytokine response scores were dramatically increased in male patient Mo/DCs relative to those of female patients, they were only modestly increased in obese patient Mo/DCs relative to patients with healthy BMIs. Accordingly, relative IFN activation scores were attenuated in obese patients more than in male patients, with only IFN-γ emerging from the upstream regulator analysis of BMI Mo/DC DEGs (Supplemental Figure 2E) and with a much lower activation score than in the analysis of sex Mo/DC DEGs (Supplemental Figure 2B). The attenuation of the IFN signal in obese patients may reflect baseline differences in obese individuals. A previous study found that healthy obese patients showed globally reduced expression of ISGs across both classical and nonclassical circulating Mos (50). This was reported to contribute to transcriptional skewing of Mos toward a regulatory phenotype that also involved increased expression of immunoregulatory molecules such as *CD52* (50), one of the top markers for obese Mo/DCs. Thus, it is possible that the variable immune cell transcriptional signatures of higher risk subgroups during COVID-19 infection result from preexisting immunological differences across patient demographics. Nonetheless, these differences may still shape patient responses to COVID-19 infection and immune-targeting therapeutics.

Our study has several limitations. First, the immune cell transcriptional shifts described here cannot be linked to differences in clinical outcomes given our intentional selection of patients with similar disease severity at hospitalization. It is possible that these shifts reflect differences between risk subgroups that render patients in the higher risk subgroup more susceptible to severe disease; conversely, it is also possible that these shifts reflect protective immune cell transcriptomic signatures that prevented the higher risk patients from presenting with critical disease. Stratified analyses of larger data sets with greater variation in clinical outcomes may clarify whether these demographic factor-dependent signatures are associated with or contribute to disease risk or protection. Second, our cohort likely included patients in different stages of COVID-19 disease. Variation in representation of disease stages across the risk factor subgroups could contribute to the observed transcriptomic differences. Third, without a similarly sized cohort of patients hospitalized for hypoxemic non–SARS-CoV-2 pneumonia for comparison, we cannot determine whether the transcriptomic signatures observed here may be specific to COVID-19 or common to patients hospitalized with respiratory viral infection. Similarly, without a comparable number of controls, we cannot ascertain whether these signatures are also present in healthy controls, reflecting baseline immunological differences. These represent important future directions for increasing understanding of immunological variation across demographic risk groups in disease and health to support health equity in future therapeutic development and clinical trial design. Fourth, our COVID-19 cohort contained only 3 individuals who self-identified as being of the Black race and 1 individual who self-identified as being of the Asian race. No patients identified as Hispanic. Black race and Hispanic ethnicity are associated with more severe COVID-19 outcomes (23). As our cohort contained several Black individuals, we adjusted for Black race in our DEG analyses; however, our study was not powered to examine transcriptomic differences across racial or ethnic groups. This is another important question meriting further investigation in future studies. Finally, our study was not designed to evaluate or account for reported interactions among the covariates examined here (51–54). These limitations notwithstanding, the findings presented here provide potentially novel transcriptomic evidence that COVID-19 immune cell responses vary across key patient demographic groups and support inclusion of a diverse patient population in COVID-19 therapeutic trials.

## Methods

### Participant enrollment.

A total of 38 patients presenting to the University of Michigan hospital with COVID-19 and 3 healthy controls were enrolled in the study between July 2020 and January 2021. Recruitment occurred within a larger clinical trial (ClinicalTrials.gov NCT04391179); however, all data presented in this study were generated or collected prior to clinical trial intervention of administration of study drug or placebo. Enrollment occurred within 1 day of hospital admission. Patients were eligible to participate if they were hospitalized for hypoxic pneumonia with confirmed SARS-CoV-2 infection by a PCR-based assay and were requiring supplemental oxygen. Patients were not eligible if they were already requiring invasive mechanical ventilation, receiving a cytokine inhibitory therapy (targeting IL-6, IL-6R, IL-1, or Janus kinase), hypotensive with systolic blood pressure less than 90 mmHg, pregnant or breastfeeding, receiving dual antithrombotic therapy (such as the combination of aspirin or P2Y12 inhibitor in addition to therapeutic anticoagulation), or classified as having had recent major bleeding defined in accordance with the criteria of the International Society on Thrombosis and Hemostasis. Exclusionary lab testing included aspartate aminotransferase or alanine aminotransferase greater than 5 times the upper limit of normal, hemoglobin less than 8 g/dL, or platelets less than 50,000 per mm. Receipt of dexamethasone prior to enrollment was not an exclusion criterion; 17 of 38 patients (45%) received dexamethasone the day before enrollment, and 29 of 38 patients (76%) received dexamethasone on the day of enrollment. Detailed patient characteristics, including demographics, laboratory data on day of enrollment, data related to oxygen requirement on day of enrollment, comorbidities and past medical history extracted from the electronic medical record, home medications extracted from the medical record, and information on the receipt of dexamethasone on the day before and day of enrollment, are available in Supplemental Table 1. ICD-10 codes used for extraction of comorbidities and past medical history are included in Supplemental Table 4.

### Leukocyte isolation for scRNA-Seq.

Whole blood was collected on the day of enrollment and buffy coats were isolated as follows: Blood was collected in sodium citrate tubes and centrifuged at 400*g* for 10 minutes at room temperature with the brake off. The buffy coat, visualized as a layer between the plasma and red blood cells, was transferred to a separate 50 mL conical. For red blood cell lysis, 20 mL of 0.2% NaCl was added. After a 45- to 60-second incubation, 30 mL of 1.8% NaCl was added. Cells were washed in PBS and then resuspended in PBS at a concentration of 1 × 10^6^ cells/mL for library preparation. All centrifugations were performed at room temperature.

### ScRNA-Seq and data analysis.

scRNA-Seq libraries were prepared by the University of Michigan Advanced Genomics Core using the Chromium single cell 3’ library and gel bead kit v3 (10x Genomics). Sequencing was performed on Illumina NovaSeq 6000 sequencer to generate 151 bp paired-end reads. Data processing, including quality control, read alignment, and gene quantification, was conducted using the Cell Ranger pipeline (10x Genomics). The digital expression matrix was analyzed using the R package Seurat (version 3.0.2). The Seurat function NormalizeData was used to normalize the raw counts and conduct further quality control, and variable genes were identified using the FindVariableGenes function. Cells with fewer than 500 UMIs, fewer than 100 genes, and greater than 10% mitochondrial expression were removed from further analysis. The ScaleData function was used to scale and center expression values in the data set for dimensional reduction. Principal component analysis and then UMAP were used to reduce the dimensions of the data, and the first 2 dimensions were used in plots. We conducted batch correction (21) and clustered cells mapped to corresponding cell types by matching cell cluster gene signatures with putative cell type–specific markers. UMAP plots of each leukocyte type colored by donor are shown in Supplemental Figure 5.

We then performed subclustering on the neutrophil, Mo/DC, T/NK cell, and B cell populations. The newly calculated subclusters were labeled using a marker gene list. Cell counts for each donor for each labeled cell subset are shown in Supplemental Table 5. DEGs were detected using the MAST approach (55). Each differential expression comparison was conducted within the cell type of interest individually, adjusting for the following covariates: age, sex, race, and BMI. For risk factor subgroup analyses, we did not adjust for the risk factor of interest and adjusted only for the 3 other covariates. Age values were sorted into the aged (≥ 65) and young (< 65) categories, and BMI was sorted into the underweight (< 18.5), normal weight (18.5–24.9), overweight (25–29.9), and obese (≥ 3 0.0) categories. Bonferroni correction was used for multiple testing, and genes with adjusted *P* values less than or equal to 0.05 and with a greater than or equal to 1.2-fold change were declared significant.

### Upstream regulator analysis.

DEG lists were filtered to exclude gene names starting with RPS, RPL, or MT, as ribosomal and mitochondrial tended to vary en bloc. IPA (QIAGEN) was used to analyze filtered DEG lists.

### Statistics.

Methods of statistical analysis are specified in the Seurat functions as listed above. For differential expression comparisons, significance was determined utilizing adjusted *P* values less than or equal to 0.05 with Bonferroni correction and a greater than or equal to 1.2-fold change. For upstream regulator analysis, a *z* score of greater than or equal to |2| was defined as significant.

### Study approval.

The University of Michigan IRB approved the protocol (IRB HUM00179783). The study was conducted in accordance with the amended Declaration of Helsinki. Written informed consent was obtained from all patients and controls.

### Data availability.

scRNA-Seq data are available at National Center for Biotechnology Information Gene Expression Omnibus GSE236177. Values for all data points found in graphs are in the Supporting Data Values file.

## Author contributions

ACB, RW, LCT, JEG, JSK, and YK designed the study. SY, CKH, and YZ conducted the experiments. YZ, NS, CP, CA, AH, MFL, KS, KR, HBI, CFB, JSK, and YK acquired the data. RW, FM, MTP, and LCT analyzed the data. JMK, JSK, and YK provided the reagents. ACB, RW, LCT, JEG, JSK, and YK wrote the manuscript with input from all other authors.

## Supplementary Material

Supplemental data

Supplemental tables 1-5

Supporting data values

## Figures and Tables

**Table 1:**
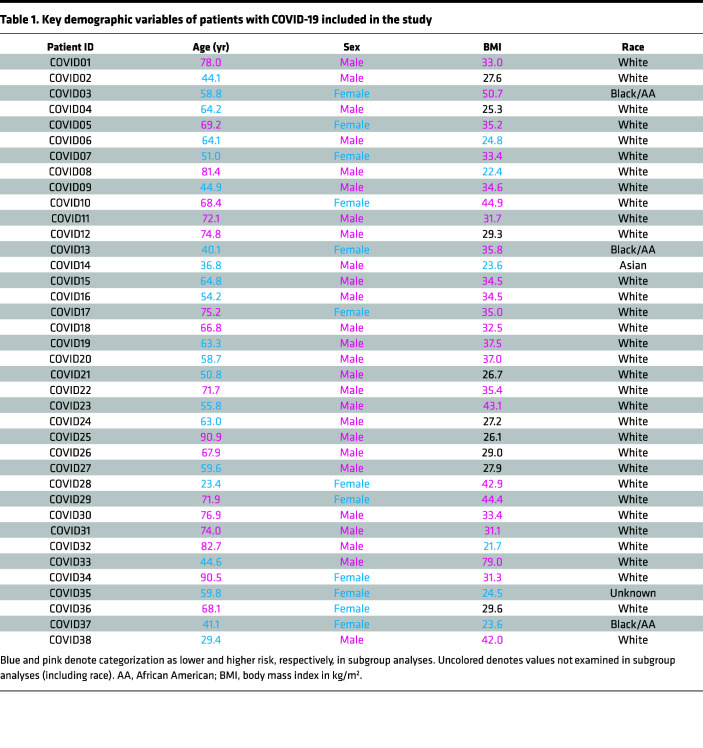
Key demographic variables of patients with COVID-19 included in the study

**Table 2:**
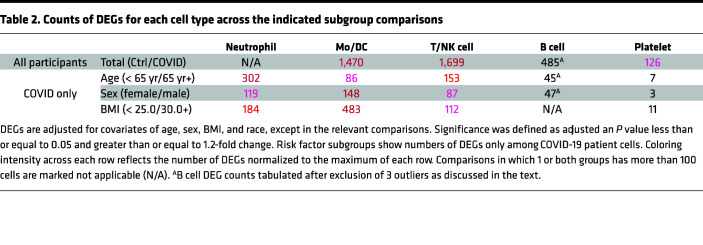
Counts of DEGs for each cell type across the indicated subgroup comparisons

## Version 1 08/22/2023

Electronic publication
