# Supplementary material for: Key patient demographics shape innate immune topography in noncritical hypoxic COVID-19 pneumonia
Source: JCI Insight. 2023 Aug 22;8(16):e166110. doi: 10.1172/jci.insight.166110 (PMC10543737; doi:10.1172/jci.insight.166110)
Supplement: Supplemental data [file jciinsight-8-166110-s202.pdf]

TITLE: Key patient demographics shape innate immune topography in non-critical hypoxic COVID-19 pneumonia

AUTHORS: Allison C. Billi[1], Rachael Wasikowski[1], Feiyang Ma[2], Srilakshmi Yalavarthi[2], Claire K. Hoy[2], Yu Zuo[2], Matthew T. Patrick[1], Neha Shah[3], Christine Parker[4], Chad Aaronson[2], Alyssa Harbaugh[2], Matthew F. Lucido[2], Kerby Shedden[2], Krishna Rao[5], Heidi B. IglayReger[4,6], Charles F. Burant[4,6,7], J. Michelle Kahlenberg[1,2], Lam C. Tsoi[1,8,9], Johann E. Gudjonsson[1]*, Jason S. Knight[2]*, Yogendra Kanthi[3,10]*

1. Department of Dermatology, University of Michigan, Ann Arbor, Michigan, USA.
2. Division of Rheumatology, Department of Internal Medicine, University of Michigan, Ann Arbor, MI, USA.
3. Division of Cardiovascular Medicine, Department of Internal Medicine, University of Michigan, Ann Arbor, MI, USA.
4. A. Alfred Taubman Medical Research Institute, University of Michigan, Ann Arbor, Michigan, USA.
5. Division of Infectious Disease, Department of Internal Medicine, University of Michigan, Ann Arbor, MI, USA.
6. Department of Internal Medicine, University of Michigan, Ann Arbor, MI, USA.
7. Department of Nutritional Sciences, University of Michigan, Ann Arbor, Michigan.
8. Department of Computational Medicine and Bioinformatics, The University of Michigan, Ann Arbor, MI, USA.
9. Department of Biostatistics, University of Michigan, Ann Arbor, MI, USA.
10. Laboratory of Vascular Thrombosis and Inflammation, National Heart, Lung, and Blood Institute, NIH, Bethesda, MD, USA.

*** Corresponding authors.**

Yogendra Kanthi:
Mailing address: Building 10, Clinical Center, Rm 5-5130, 10 Center Drive, Bethesda, MD 20892, USA
Telephone: +1 (301) 827-3383
e-mail: yogen.kanthi@nih.gov

Jason S. Knight:
Mailing address: 1150 West Medical Center Dr., Ann Arbor, MI, 48109, USA
Telephone: +1 (734) 763-3031
e-mail: jsknight@umich.edu

Johann E. Gudjonsson:
Mailing address: 1910 Taubman Center, 1500 E. Medical Center Dr., Ann Arbor, MI, 48109, USA
Telephone: +1 (734) 936-4054
e-mail: johanng@med.umich.edu

CONFLICT OF INTEREST: KR is supported in part from an investigator-initiated grant from Merck & Co, Inc.; he has consulted for Seres Therapeutics, Inc., Rebiotix, Inc. and Summit Therapeutics, Inc. YK is an inventor on a patent application (US20220160756A1) filed by the University of Michigan on the use of biogases for the treatment of vascular disease.

SUPPLEMENTAL MATERIAL:

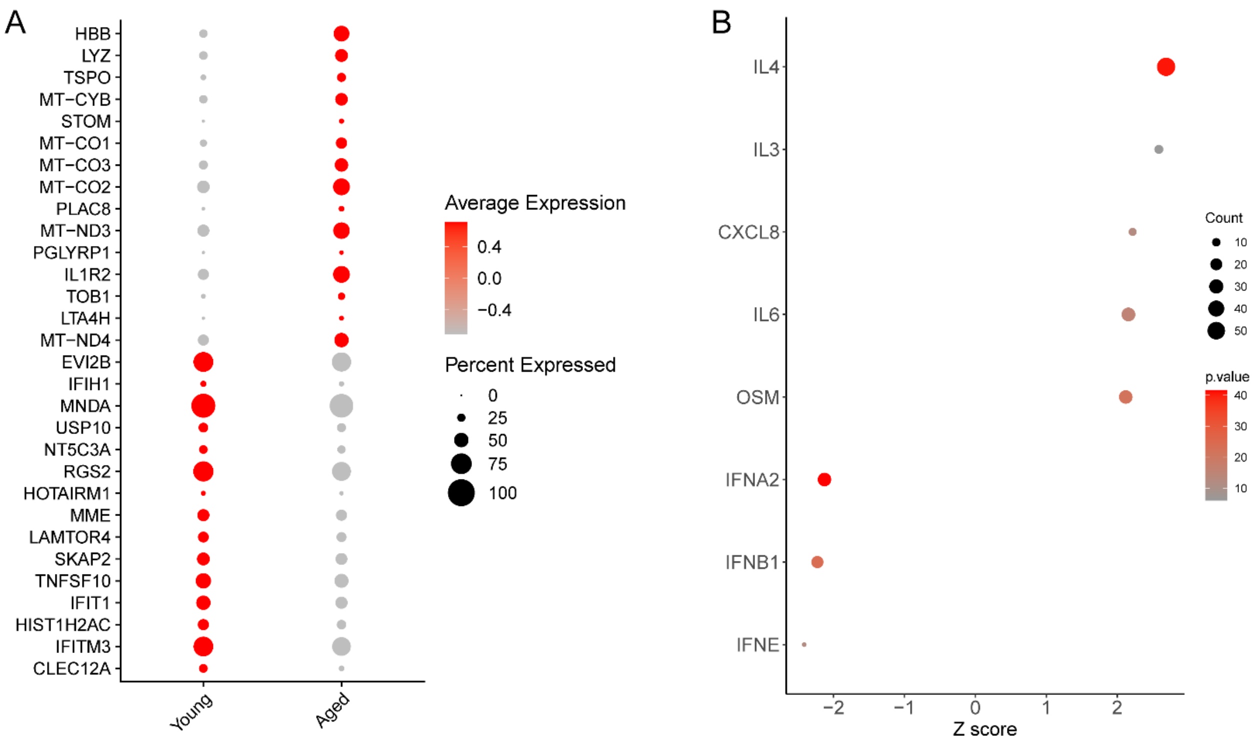

Supplemental Figure 1. Neutrophil DEGs reveal dysregulated interferon (IFN) responses in COVID-19 age comparison. A. Top differentially expressed genes (DEGs) in neutrophils from aged (65 years of age and older) and young (<65 years of age) COVID-19 patients ranked by fold change. **B.** Upstream regulators significantly (|z-score|≥2) activating the aged transcriptional signature in COVID-19 patient neutrophils ranked by z-score (positive, activated in aged; negative, inhibited in aged).

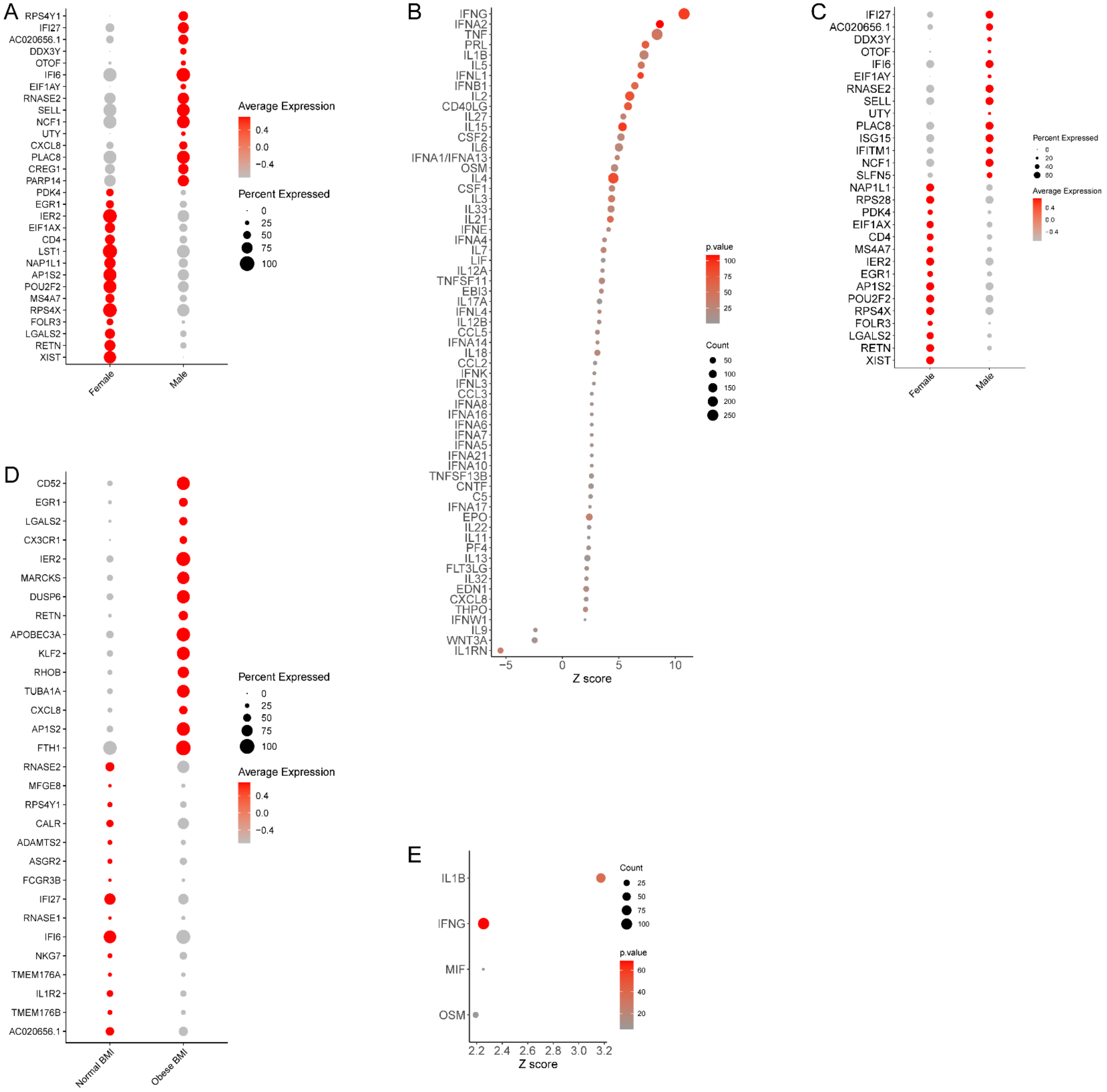

Supplemental Figure 2. Monocyte/dendritic cell DEGs reveal distinct transcriptional COVID-19 risk signatures. A. Top 15 DEGs in Mo/DCs from male and female COVID-19 patients ranked by log(fold change). **B.** Upstream regulators significantly activating and inhibiting the male-biased transcriptional signature in COVID-19 patient Mo/DCs ranked by z-score (positive, activated in male; negative, inhibited in male). **C.** Top 15 DEGs in CD14+ monocytes from male and female COVID-19 patients ranked by log(fold change). **D.** Top 15 DEGs in Mo/DCs from COVID-19 patients with obese and normal body mass index (BMI) patients ranked by log(fold change). **E.** Upstream regulators significantly activating and inhibiting the obese BMI-biased transcriptional signature in COVID-19 patient Mo/DCs ranked by z-score (positive, activated in obese; negative, inhibited in obese).

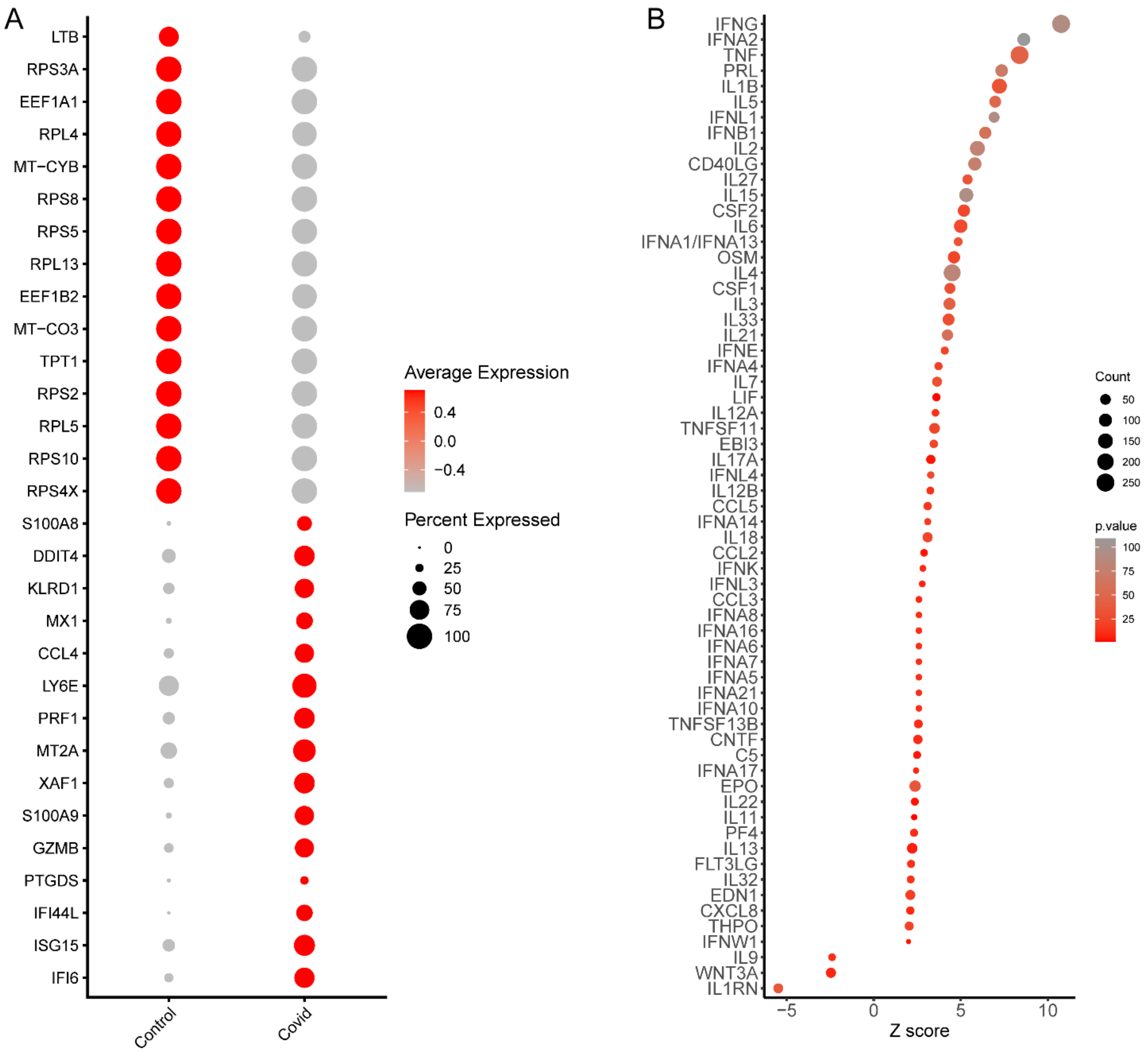

Supplemental Figure 3. T/NK cell DEGs show increased ISGs, cytotoxic granular factors, and S100 genes in COVID-19 versus control patient comparison. A. Top differentially expressed genes (DEGs) in T/NK cells from COVID-19 versus control patients ranked by fold change. **B.** Upstream regulators significantly ($|z| \geq 2$) activating the COVID-19 disease transcriptional signature in T/NK cells ranked by z-score (positive, activated in COVID-19 patients; negative, inhibited in COVID-19 patients).

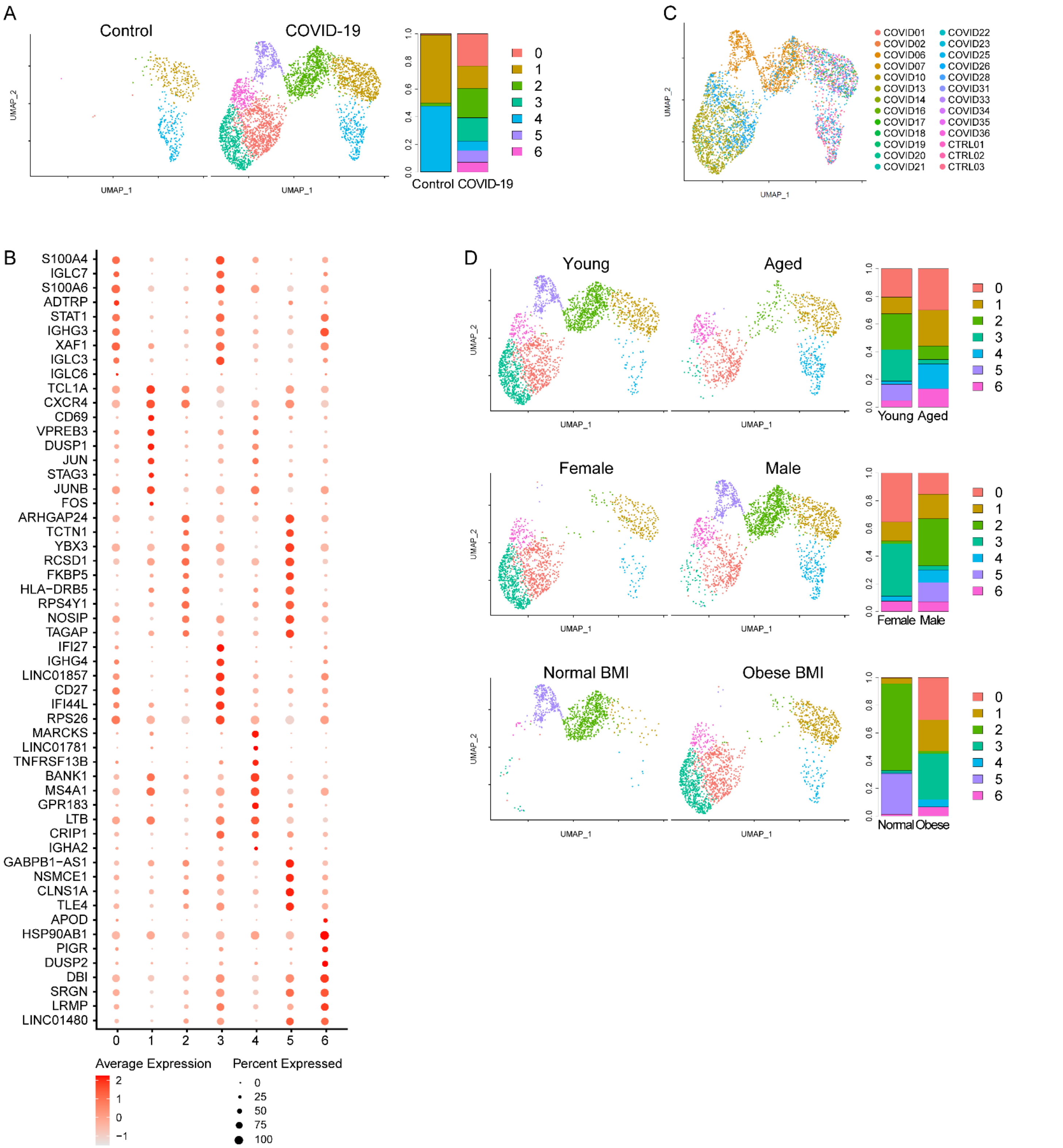

Supplemental Figure 4. B cell sub-clustering identifies three COVID-19 patients with B cell expansion. A. UMAP plot of 4,247 B cells colored by sub-cluster and split by disease state. Bar plot, proportion of cells in each sub-cluster split by disease state. **B.** Dot plot of representative marker genes for each B cell sub-cluster. Color scale, average marker gene expression. Dot size, percentage of cells expressing marker gene. **C.** UMAP plot of B cells colored by donor. **D.** UMAP plots and bar plots of B cells split by risk factor subgroup. Only COVID-19 patient cells from the indicated risk factor subgroups are presented on each plot.

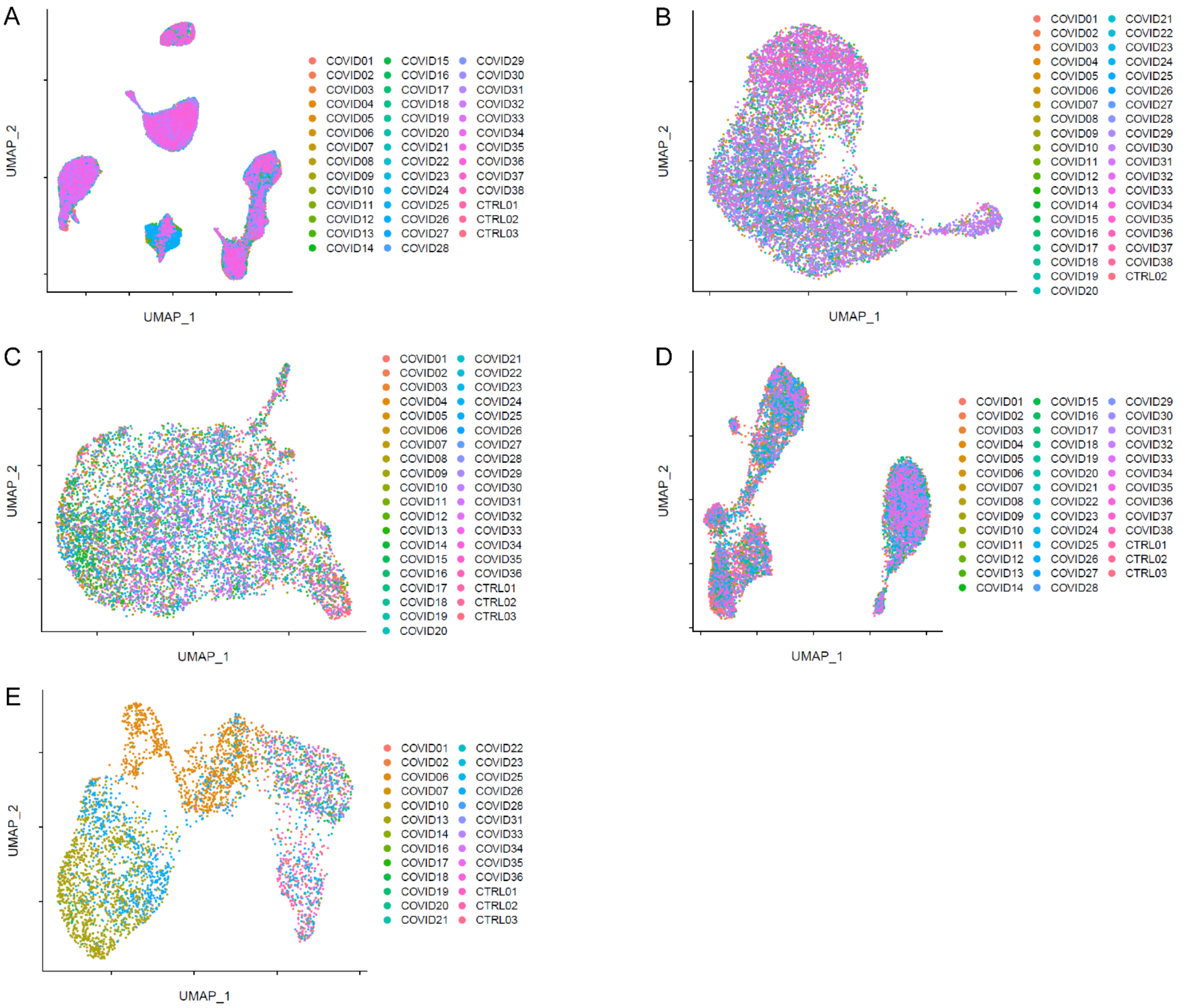

Supplemental Figure 5. UMAP plots of each leukocyte type colored by donor. The B cell panel is replicated as **Supplemental Figure 4C**.

[See .xlsx file]

Supplemental Table 1. COVID-19 patient characteristics. For comorbidity and past medical history data as well as dexamethasone therapy data, a value of 0 indicates no and a value of 1 yes. ICD-10 codes extracted for comorbidity and past medical history data are available in **Supplemental Table 4**.

		Neutrophil	Monocyte/DC	T/NK cell	B cell	Platelet
	Control	45	952	2920	438	439
COVID-19 patients	COVID-19	10978	7468	7919	869	964
	Young	5720	3685	3867	416	593
	Aged	5258	3783	4052	453	371
	Female	3830	2333	2573	266	452
	Male	7148	5135	5346	603	512
	Normal BMI	1616	1235	564	50	124
	Obese BMI	6989	4850	4914	532	634

Supplemental Table 2. Cell counts for the indicated subgroups within each cell type. Grey shading indicates comparisons lacking an adequate cell count (<100 cells for either subgroup) for DEG calculation. Control patient cells are represented only in the Control row. All other counts represent only COVID-19 patient cells (indicated by the thick border). B cell values reflect counts after exclusion of three outliers as discussed in the text. DC, dendritic cell; NK, natural killer.

[See .xlsx file]

Supplemental Table 3. Differentially expressed genes (DEGs) for each cell type across the indicated comparisons. Risk factor subgroups show numbers of DEGs only among COVID-19 patient cells. DEGs are adjusted for covariates of age, sex, BMI, and race (excluding the covariate being examined).

[See .xlsx file]

Supplemental Table 4. ICD-10 codes used for extraction of comorbidity and past medical history data in Supplemental Table 1.

[See .xlsx file]

Supplemental Table 5. Cell counts for each donor for each labeled cell type.
